# Interplay Between the Immune and Endocrine Systems in the Lung: Implications for TB Susceptibility

**DOI:** 10.3389/fimmu.2022.829355

**Published:** 2022-02-22

**Authors:** Tariq Webber, Katharina Ronacher, Marli Conradie-Smit, Léanie Kleynhans

**Affiliations:** ^1^DSI-NRF Centre of Excellence for Biomedical Tuberculosis Research, South African Medical Research Council Centre for Tuberculosis Research, Division of Molecular Biology and Human Genetics, Department of Biomedical Sciences, Faculty of Medicine and Health Sciences, Stellenbosch University, Cape Town, South Africa; ^2^Translational Research Institute, Mater Research Institute – The University of Queensland, Brisbane, QLD, Australia; ^3^Division of Endocrinology, Department of Medicine, Faculty of Medicine and Health Sciences, Stellenbosch University, Cape Town, South Africa

**Keywords:** immuno-endocrine interactions, hormone, type 2 diabetes, tuberculosis, hormone receptor

## Abstract

The role of the endocrine system on the immune response, especially in the lung, remains poorly understood. Hormones play a crucial role in the development, homeostasis, metabolism, and response to the environment of cells and tissues. Major infectious and metabolic diseases, such as tuberculosis and diabetes, continue to converge, necessitating the development of a clearer understanding of the immune and endocrine interactions that occur in the lung. Research in bacterial respiratory infections is at a critical point, where the limitations in identifying and developing antibiotics is becoming more profound. Hormone receptors on alveolar and immune cells may provide a plethora of targets for host-directed therapy. This review discusses the interactions between the immune and endocrine systems in the lung. We describe hormone receptors currently identified in the lungs, focusing on the effect hormones have on the pulmonary immune response. Altered endocrine responses in the lung affect the balance between pro- and anti-inflammatory immune responses and play a role in the response to infection in the lung. While some hormones, such as leptin, resistin and lipocalin-2 promote pro-inflammatory responses and immune cell infiltration, others including adiponectin and ghrelin reduce inflammation and promote anti-inflammatory cell responses. Furthermore, type 2 diabetes as a major endocrine disease presents with altered immune responses leading to susceptibility to lung infections, such as tuberculosis. A better understanding of these interactions will expand our knowledge of the mechanisms at play in susceptibility to infectious diseases and may reveal opportunities for the development of host-directed therapies.

## Introduction

There is a growing interest in the relationship between the immune and endocrine systems (1). Bidirectional communication between the immune and endocrine systems facilitates optimal host responses during infection and homeostasis. This communication is possible since endocrine organs express cytokine receptors and cells of the immune system express hormone receptors (2). Metabolic, nutritional, psychosocial, and genetic factors influence the immune response *via* endocrine signaling. During infection, cytokines produced by immune cells directly or indirectly alter hormonal responses, developing a feedback response between the endocrine and immune systems. Cytokines induce alterations in hormone production, by directly affecting endocrine organ function, such as IL-1, IL-6, and TNF-α. These cytokines increase the activity of the hypothalamus-pituitary-adrenal (HPA) and hypothalamus-pituitary-thyroidal (HPT) axes, and reduce that of the hypothalamus-pituitary-gonadal (HPG) axis (3–6), or indirectly by promoting cellular destruction of endocrine cells, as in the pancreatic dysfunction of type 2 diabetes (T2D) (7).

Hormones exert a profound effect on the functions of the immune system, exemplified by the anti-inflammatory functions of glucocorticoids (GCs) and androgens (8). While acute stress may be beneficial for the immune system during fight or flight, chronic stress is harmful. Chronic inflammation signal cortisol release through inflammatory signaling as well as the HPA axis and can drive HPA axis dysfunction if left unchecked. Stress-induced activation of the HPA axis results in the secretion of corticotropin releasing hormone (CRH) from the hypothalamus, which stimulates the production of adrenocorticotropic hormone (ACTH) from the anterior pituitary gland. ACTH stimulates the adrenal glands to produce cortisol, a potent anti-inflammatory hormone. To limit long-term exposure of tissues and cells to the immunosuppressive actions of cortisol, a negative feedback loop allows cortisol to regulate its own secretion. Long-term exposure to elevated levels of cortisol may lead to adaptations in HPA axis function, resulting in an initial increase in cortisol production (hypercortisolism) followed by a decrease in the production of the hormone (hypocortisolism) or cortisol resistance (9). Thus, chronic inflammatory responses and a dysregulated HPA axis result in altered cytokine signals and immune cell function (10).

Type 2 diabetes is a metabolic disease that presents with numerous alterations in hormone levels, including increased insulin and cortisol levels, and reduced free triiodothyronine (fT3) and testosterone levels (11–14) (**Table 1**). Common comorbidities of T2D include depression, asthma, and chronic obstructive pulmonary disease, which are co-prevalent and may indicate a relationship between stress-related hormonal alterations and lung function, as well as hypothyroidism (34). Altered endocrine responses in T2D are, in part, related to dysregulation of the HPA, HPT, and HPG axes, having downstream effects on immune functions within various organs (35–37).

**Table 1:** Inflammatory Properties of hormones and their relative levels measured in the periphery.

Function in lung	Hormone	T2D (vs healthy)	In TB (vs healthy)	TB-T2D (vs TB)
Anti-inflammatory	CRH	Low (15)		
	ACTH	High (15)		
	Cortisol	High (15)	High (3, 16)	
	T4	No change (17)	High (3)	High (18)
	Testosterone	Low (19)	Low (3), No change (18)	No change (18)
	Estradiol	Low (20)	High (3, 21), No change (18)	High (18)
	Adiponectin	Low (22)	No change (16)	Low (23)
	GIP	Low (24)		
	GLP-1	Low (24)		
	Ghrelin	Low (25)	Low (26), No change (16), High (27)	No change (26)
	Glucagon	High (28)		
	C-peptide	High (29)		
Pro-inflammatory	TSH	No change (17)		
	Lipocalin-2	Low (22)		
	DHEA	Low (30)	Low (3, 16)	High (31), No change (18)
	Leptin	High (22)	Low (16, 27), High (26)	Low (26), High (23)
	Resistin	High (22)	High (27)	
	Insulin	High (32, 33)		
	Growth Hormone	Low (33)	High (3, 18)	High (31), No change (18)

T2D, Type 2 Diabetes; TB, Tuberculosis; CRH, corticotropin-releasing hormone; ACTH, adrenocorticotropic hormone; T4, Thyroxine; GIP, gastric insulinotropic peptide; GLP-1, glucagon-like peptide 1; TSH, thyroid-stimulating hormone; DHEA, dehydroepiandrosterone.

**Figure 1:**
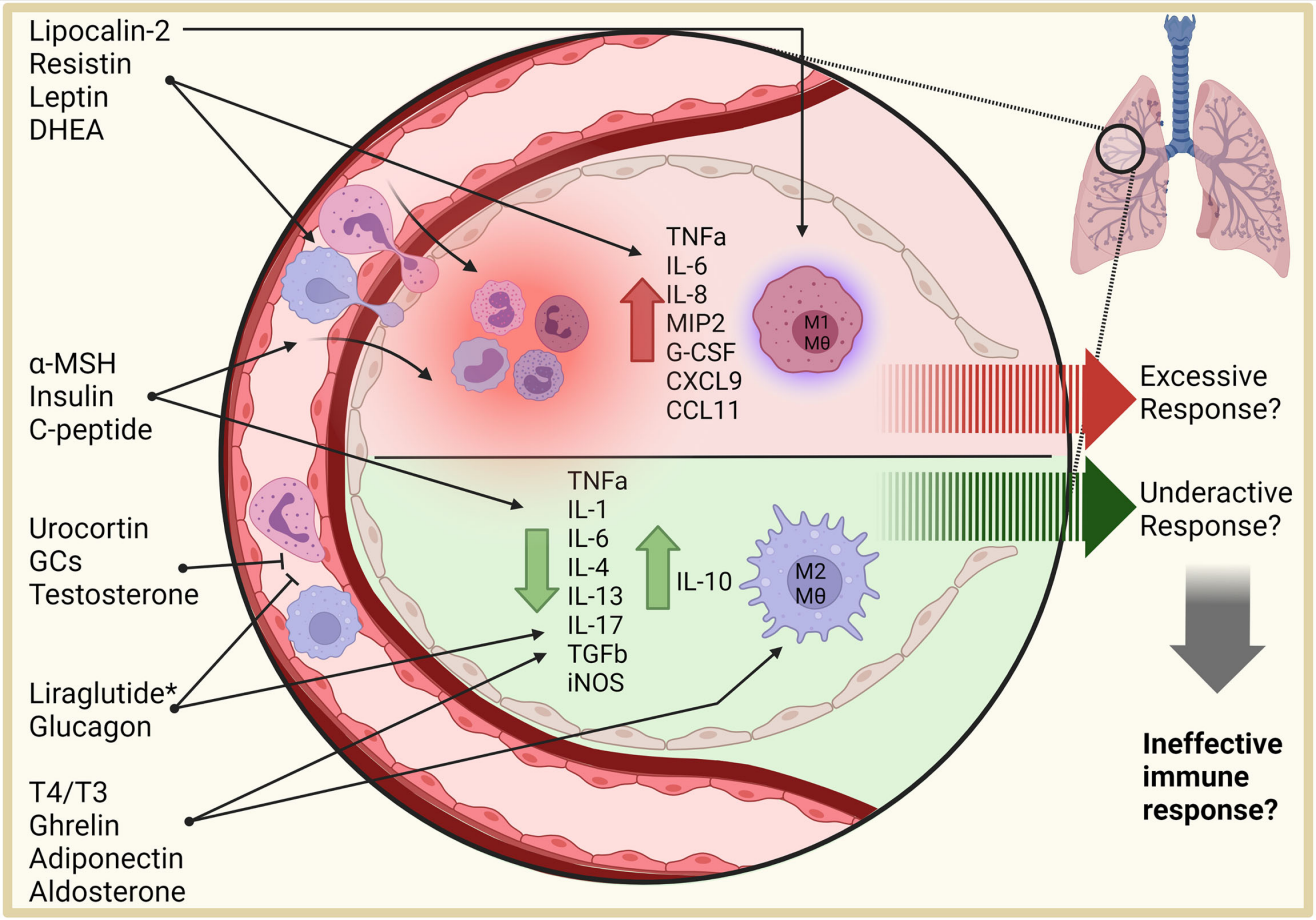
Effects of hormones on the lung environment. Hormones influence inflammatory responses, necessitating a clear understanding of their role in infectious disease. Pro-inflammatory adipokines, such as resistin, leptin, lipocalin-2, and DHEA collectively stimulate migration of immune cells into the lung and promote a pro-inflammatory signaling environment, with lipocalin-2 stimulating macrophage activation and M1 polarization. Anti-inflammatory hormones, including glucocorticoids, testosterone, glucagon, thyroxine, ghrelin, adiponectin, and aldosterone, reduce inflammatory signaling, immune cell recruitment, and polarize macrophages to anti-inflammatory phenotypes. Whether these combined effects are beneficial or detrimental to infectious disease response in the lung require further investigation. Additionally, agonists and antagonists (*) provide a future opportunity to regulate these processes. DHEA, dehydroepiandrosterone; α-MSH, alpha-melanocyte-stimulating hormone; T4, Thyroxine; T3, triiodothyronine; Mθ, macrophage. This figure was created with BioRender.com.

Although the lungs are a major site of acquired infections, the effects of hormonal dysregulation on the immune response within the lung are still largely unknown. This review discusses hormones with immune modulatory potential in the lung and their influence on the immune response therein (summarized in **Figure 1**). Implications for susceptibility to tuberculosis (TB) are highlighted, drawing on examples of endocrine dysregulation in the form of T2D.

## Central Endocrine (Hypothalamic and Pituitary) Hormones

### Corticotropin Releasing Hormone (CRH)

CRH is primarily produced by the hypothalamus, but can also be expressed at peripheral sites where it acts as an autocrine or paracrine inflammatory modulator (38, 39). CRH binds with a high affinity to CRH receptor 1 (CRHR1) and CRH-like peptides with a high affinity to CRHR2. CRHR2 is the major receptor expressed in human lungs, however compared to other tissues (heart, stomach, liver and adrenal), the lung and skeletal muscle express the highest levels of CRHR1 (40). In mice, the CRHR1/R2 ratio changes in response to stress in different immune cell types in the lung (41). CRHR1 mRNA increases and CRHR2 decreases in dendritic cells, whereas CRHR2 increases in macrophages in response to stress. Furthermore, CRHR2 is found to be preferentially expressed in neutrophils in the lungs of mice (42).

Differential expression of CRHR1 and CRHR2 in phagocytic cells in the lungs could affect innate cell function, potentially leading to altered adaptive immune responses. Limited information is available on how targeting CRH and CRHR activity in the respiratory tract impacts bacterial disease outcome. In a murine respiratory *Streptococcus pneumoniae* infection model, Burnley and Jones (2017) demonstrated that intranasal CRH administration reduces the pulmonary inflammatory response, and the subsequent recruitment of neutrophils and monocytes to the lung, improving survival in these animals (43). Moffat et al. (2006) demonstrated that, urocortin, a structurally related peptide to CRH, causes bronchorelaxation and limits lipopolysaccharide (LPS)-induced pulmonary inflammation (specifically neutrophil recruitment) to a greater extent than CRH (44). During *S. pneumoniae* infection in the lungs of CD-1 mice, an outbred strain of albino mice, prior CRHR2 antagonist (astressin 2B) treatment reduces pulmonary bacterial growth and prevents sepsis, while CRHR1 antagonism (antalarmin) provided no benefit (39). Stimulation of CRHR in the lung, therefore, provides a promising approach for reducing excessive inflammation in the lungs and should be studied further.

### Adrenocorticotropic Hormone (ACTH)

ACTH is a pituitary hormone that signals *via* melanocortin receptors (MCRs), of which there are five. They are expressed on a wide range of tissues, including nervous, intestinal, adipose, skeletal, and lung tissues. Among these receptors, MC1R and MC5R are expressed in lung tissues. MC1R, MC3R, and MC5R are expressed on macrophages and lymphocytes, while MC2R and MC4R are expressed on lymphocytes (45). Cleavage of the first 13 amino acids of ACTH yields alpha melanocyte-stimulating hormone (α-MSH).

ACTH and α-MSH exert broad anti-inflammatory effects in a variety of tissues by signaling through the MCRs, with the major receptor exerting these effects being MC1R (45). Additionally, numerous anti-inflammatory melanocortin-based therapies are in clinical trials for the treatment of inflammatory conditions (46). In allergic mice, intraperitoneal α-MSH reduced peribronchial airway inflammation, altering leukocyte populations in bronchoalveolar lavage (BAL) fluid, and reduced IL-4 and IL-13 levels, effects dependent on IL-10 signaling downstream of α-MSH treatment (47). In acute lung injury mouse models, α-MSH treated mice displayed reduced edema; IL-6, TNF-α, and TGF-β gene expression; and leukocyte infiltration in the lung (48, 49). Thus, MCR stimulation in the lung may provide a novel target for its anti-inflammatory properties, providing repurposing potential for current agonists used to treat obesity (setmelanotide), porphyria (afamelanotide), and reduced sexual desire in women (bremelanotide) (50).

### Growth Hormone-Releasing Hormone (GHRH) and Growth Hormone (GH)

GHRH is secreted predominantly by the hypothalamus and stimulates the secretion of GH by the pituitary gland and regulates hepatic insulin-like growth factor-1 (IGF-1) production through the GH/hepatic IGF-1 axis (51). Other tissues, including the lungs also produce GHRH locally and express the receptor of GHRH (GHRHR) as well as the GHRHR splice variant 1 (SV1) (52). In normal mouse lung tissue, type 2 cells, club cells and fibroblasts, lymphocytes, and dendritic cells express GHRH (52). GHRH is involved in lung homeostasis, inflammation and fibrosis (51). Antagonists for GHRHR have anti-inflammatory, pro-apoptotic, anti-oxidative and anti-fibrotic effects in the lung. The GHRH antagonist MIA-602, frequently used in bleomycin-induced lung fibrosis models, suppresses ERK1/2 and JAK2/STAT3 pathway activation and increases P53 and pAMK levels (53). At mRNA level MIA-602 downregulates pathways involved in adaptive immune responses including T cell activation and differentiation, T cell signaling and cytokine production (54). In a sarcoidosis granuloma model, GHRHR inhibition results in reduced IL-12 and IL-17A production (54, 55). Thus, MIA-602 demonstrates the contribution of GHRHR to adaptive immunity and T cell function, and that GHRHR is a potential candidate for managing these responses when they are excessive, such as in granulomatous diseases.

The ability of GHRHR signaling to modulate lung immune responses was further demonstrated in a study assessing the vaccine-induced immune responses of GHRH knock-out mice against *S. pneumoniae*. Knock-out mice were significantly more susceptible to infection after vaccination than wild-type mice, with vast infiltration of neutrophils and monocytes, and reduced B cells and T cells in the lung post infection (56). These knock-out mice were unable to mount a pneumococcal vaccine-induced response, a deficiency restored by GH treatment, highlighting the importance of GH signaling in the lung immune response. The pro-inflammatory characteristics of GH have been demonstrated by exacerbating LPS-induced inflammation and cecal ligation and puncture (CLP)-induced sepsis by increasing neutrophil NF-κB activity, neutrophil presence, microvascular injury, and neutrophil CD11b expression in the lungs of rats (57, 58). In contrast, others have found GH treatment to prevent acute lung injury by reducing ICAM-1 expression and NF-κB activity in lungs (59, 60). The two studies displaying the pro-inflammatory effects of GH administered the GH subcutaneously to male Wistar rats, while the latter studies observing anti-inflammatory effects administered the GH intramuscularly in Sprague-Dawley rats and pathogen-free Kun Ming mice, respectively. Considering endocrine differences between Wistar and Sprague-Dawley rats (61), hormone-induced responses may differ and validation of observations across model organisms is key for avoiding model specific interpretations.

## Adrenal Hormones

### Glucocorticoids (GCs) and Mineralocorticoids

Cortisol is a known immune modulator and synthetic GCs have been used clinically to control inflammatory conditions, such as asthma, for decades. Cortisol is produced from cholesterol that is converted to pregnenolone before the steroidogenic pathway diverges toward the formation of this major class of steroid hormones. It signals *via* the glucocorticoid receptor (GR) and mineralocorticoid receptor (MR), which are ubiquitously expressed, including in the lung (62).

High doses of GCs reduce the number of macrophages in the respiratory tract and impair the functional activity of resident lung macrophages (63). GCs are produced in T-cells and macrophages of the lung upon stimulation with inflammatory mediators, including TNF-α, LPS, and anti-CD3, acting as an immunoregulatory mechanism to limit uncontrolled inflammatory responses in the lung. This GC stimulatory effect was promoted by intranasal leptin pretreatment in mice with LPS-induced acute lung injury (64, 65). More recently Marin-Luevano et al. (2021) demonstrated that cortisol promotes the intracellular growth of *Mycobacterium tuberculosis* in A549 type 2 pneumocytes and THP-1–derived macrophages, but not airway bronchial epithelial cells (66), highlighting the immunosuppressive effects of GCs in the lung. Long-term (>1 year) treatment with inhaled corticosteroids is implicated in increased risk of developing pneumonia (risk ratio of 1.41) and is associated with the risk of mycobacterial diseases, such as TB (67).

### Androgens

Dehydroepiandrosterone (DHEA) is produced from cholesterol, predominantly in the zona reticularis of the adrenal glands upon ACTH stimulation, and in the gonads upon luteinizing hormone and follicle-stimulating hormone stimulation. Receptors for DHEA include the androgen receptor (AR), estrogen receptor (ER), peroxisome proliferator activated receptor-alpha (PPARα), pregnane X receptor, and neurotransmitter receptors, such as the N-methyl-D-aspartate (NMDA) receptor (68, 69).

DHEA treatment is associated with reduced pulmonary hypertension by upregulating soluble guanylate cyclase and by inhibiting src/STAT3 pathway activation, a key pathway in cytokine-induced cell activation and proliferation (70, 71). Generally DHEA counteracts the effect of cortisol, and more specifically in alveolar macrophages, restores the expression of the receptor for activated C kinase (RACK-1) and LPS-induced TNF-α and IL-8 production that is reduced due to aging in rats (72). When added to alveolar macrophages from non-smoking asbestos workers *in vitro*, DHEA reduces the release of superoxide anion (73), which is consistent with its role in limiting Th2 responses (74). Intratracheal administration of 16α-bromoepiandrosterone (BEA; a DHEA-related synthetic sterol) reduces the bacterial load and inflammation in the lungs of diabetic mice who are infected with *M. tuberculosis* (75). BEA decreases the expression of the enzyme 11-β-hydroxysteroid dehydrogenase type 1, which catalyzes the conversion of inactive cortisone into active cortisol in humans and inactive dehydrocorticosterone into active corticosterone in mice. At the same time, it increases the expression of 11-β-hydroxysteroid dehydrogenase type 2, which again converts active cortisol/corticosterone into inactive cortisone/dehydrocorticosterone. This could increase IFN-γ and TNF-α response resulting in lower bacterial loads in the lungs of these animals. The ability of DHEA and BEA to improve immune and metabolic (as shown in the TB-T2D mouse model) responses suggests that they could potentially be used as therapeutic agents, which will benefit the host’s immune, endocrine and metabolic functions.

## Thyroid Hormones: Thyroxine (T4) and Triiodothyronine (T3)

The lungs express lower levels of the thyroid hormone receptors *THRA1*, *THRA2*, and *THRB1* mRNA than other tissues, however the relative protein expression of THRA2 and THRB1 in lung tissue is higher than in other human tissues (76). Furthermore, THRA expression in the lung is highest in alveolar type 1 epithelial cells, while their expression is similar in lung T cells, granulocytes, type 2 alveolar cells, and macrophages. THRB on the other hand is expressed predominantly in type 2 alveolar cells (77). In rats, thyroid hormone receptors were also found in lung type 2 alveolar cells, and promote GC responses and adenylate cyclase activity in the lung (78–80). Additionally, T3 activates the MAPK/ERK1/2 pathway in rat alveolar epithelial cells and increases their sodium-potassium-ATPase pump content and activity (81).

More recently, Ning et al. (2018) described the ability of T4 to reduce the inflammatory response and senescence in an oxidized low-density lipoprotein-induced foamy macrophage model (82). In a type 2 deiodinase-knockout mouse model, active thyroid hormones are not produced in the thyroid gland. Type 2 deiodinase-knockout mice have a greater susceptibility to ventilator-induced lung injury, with increased *Cxcl1*, *Tnf*, *Il1b*, and *Cxcl2* gene expression. Treatment with T3 ameliorated the higher cytokine and chemokine expression in knockout mice (83). Although it remains unclear whether these effects were mediated by the THR, this highlights the anti-inflammatory effects of T3 and potentiates a relevance for T3 signaling in the lung. Studies assessing the influence of thyroid hormones on lung function during infection would provide valuable information against infections.

## Gonadal Hormones

### Testosterone

Differences between male and female responses in disease are common, highlighting a potential role for sex hormones in disease. Testosterone acts *via* the AR, which is expressed in human lungs primarily in endothelial cells and club cells, fibroblasts, and type 2 alveolar cells, with lower expression in macrophages, T cells, and granulocytes (84). Testosterone exposure in mouse lungs upregulates genes involved in iron binding and oxygen transport while downregulating genes involved in DNA repair and recombination (85). In an asthma mouse model, ovalbumin sensitized male mice displayed lower susceptibility to inflammation due to lower Th2 cytokine production and lung lymphocyte levels compared to female mice (86). Becerra-Diaz et al. (2018) investigated the immune modulatory functions of AR signaling in the lungs, and found that dihydrotestosterone reconstitution in castrated mice reduced lung inflammation and enhanced M2 polarization of alveolar macrophages *via* IL-4 stimulation (87). Similarly, gonadectomized male mice infected with influenza A virus and treated orally with testosterone have reduced pulmonary monocyte and virus-specific CD8+ T cell infiltration resulting in improved disease outcomes (88). Testosterone decreases pro-inflammatory cytokine release from monocytes and macrophages, and increases the accumulation of cholesterol esters in human monocyte-derived macrophages (89). Additionally, type 2 innate lymphoid cells express AR, the signaling of which inhibits their maturation and IL-33–mediated lung inflammation.

### Estrogen

Estrogens comprise three major forms, estrone, estradiol, and estriol. Their proportions fluctuate depending on the processes occurring in the body, with estradiol predominating most of the time. During pregnancy, enzymes in the placenta transform 16α-hydroxy-dehydroepiandrosterone sulfate (16a-OH-DHEAS) to estriol. Estrone is produced mostly during menopause. Signaling of these hormones is mediated by the α and β forms of the ER. Both ER forms are expressed in human and mouse lungs, and human lung cell lines; although in humans, ER-α presence varies among individuals (90). ER-α predominates in lung fibroblasts, with low expression in endothelial cells, granulocytes and macrophages, while ER-β predominates in human lung alveolar type 1 cells, ciliated cells, and granulocytes (84). Histological analysis in CD-1^®^ IGS mouse and porcine lung show no ERα expression (91, 92), while RT-PCR in BALB/c mice shows expression of both receptors in the lung, with lower ERα expression (93). Kan et al. (2008) investigated the effect of 17β-estradiol (E2) on trauma-hemorrhage–induced lung injury in Sprague-Dawley rats. E2 administration induced higher endothelial nitric oxide synthase (eNOS) expression and phosphorylation, protein kinase G-1 activation, and VASP expression, resulting in reduced lung injury (94). These anti-inflammatory properties were also demonstrated in seawater induced acute lung injury in rats, which was reduced by E2 treatment (95). Despite ERβ predominance, ER-α is reported to be responsible for the anti-inflammatory effects of ER stimulation (96).

## Hormones of the Gastro-Intestinal Tract

### Gastric Inhibitory Polypeptide (GIP) and Glucagon-Like Peptide-1 (GLP-1)

The GIP receptor (GIPR) is present in airway ciliated and club cells, alveolar pneumocytes and lung macrophages (77). In atherosclerosis mouse models, GIP has been shown to prevent monocyte and macrophage activation (97, 98). Studies into the immune regulatory roles of GIP are scarce and may reveal additional functions of this hormone on the lung immune response.

The GLP-1 receptor (GLP-1R) is expressed in the lungs of mice and humans at a higher level than other tissues (99, 100). Numerous studies, as reviewed by Lee and Jun (2016), describe the anti-inflammatory effects of GLP-1 and the potential of GLP-1–based therapies (101). In a BALB/c mouse model of ovalbumin-induced asthma, protein kinase A (PKA) phosphorylation was inhibited and NF-κB p65 upregulated in the lung. GLP-1 agonist treatment in these mice led to PKA activation and downstream inhibition of NF-κB, mediating anti-inflammatory responses in the lung by reducing the infiltration of inflammatory cells, tissue pathology, Th2-associated cytokines in BAL fluid, and E-selectin expression (102). Gou et al. (2014) showed that bleomycin-induced pulmonary fibrosis in mice presents with an increase in macrophages and lymphocytes, as well as TGF-β1 concentrations in the BAL fluid, and VCAM-1 expression and NF-κB activation in the lung. GLP-1 agonist liraglutide, when administered intraperitoneally, reduces these characteristics of bleomycin-induced lung inflammation and fibrosis (103). Although subcutaneous liraglutide improved lung function in a chronic obstructive pulmonary disease mouse model, this effect was not accompanied by a reduction in lung inflammation. Additionally, liraglutide did not alter inflammatory cytokine responses in another study by the same group (100, 104). These differences in observations suggest that intraperitoneal administration may be required to produce the anti-inflammatory effects of GLP-1R in the lung. Other possible immunomodulatory mechanisms of GLP-1 may involve alteration of T cell function by reducing CD28 and CD86 expression, and reducing tissue factor and PAI-1 production (105). While GIPR function in the lung immune response requires further investigation, GLP-1R may reduce inflammation by mediating immune cell recruitment and activation in the lung, although it is not crucial for a balance between pro- and anti-inflammatory responses.

### Ghrelin

Since its discovery in 1999, numerous actions of ghrelin have been described ranging from metabolic homeostasis, circadian rhythms, learning and memory to immune modulation. Ghrelin binds to the growth-hormone secretagogue receptor 1a (GHSR-1a), inducing GH release from anterior pituitary cells in a process distinct from that of GHRH (106). In Hartley guinea-pigs, Wistar rats and male C57BL6/J mice, the expression of GHSR-1a mRNA was very low in the lung (107, 108), with the lung also having the highest ghrelin expression in Wistar rats (109). In two studies assessing human lungs, mRNA and protein expression of ghrelin and biologically inactive GHSR-1b was detectable, while those of GHSR-1a were not (110–112).

Despite this, ghrelin treatment is associated with alterations in inflammatory responses in lung tissues, including a reduction in inflammatory cytokine release, NF-κB pathway activation, neutrophil infiltration, and improved survival in rats with CLP-induced lung injury (113). Additionally, these anti-inflammatory effects may be related to the acylation status of ghrelin, since only acyl ghrelin and not des-acyl ghrelin binds to GHSR-1a (114). Interestingly, it has been proposed that GHSR-1a is not required for the anti-inflammatory actions of ghrelin, suggesting another as yet unknown signaling mechanism mediating these effects (115). LPS-induced apoptosis of alveolar macrophages is attenuated by ghrelin, with GHSR-1a–mediated JNK inhibition and Wnt/B-catenin activation, promoting alveolar macrophage survival and their anti-inflammatory responses. In GHRS-1a knockdown mediated by siRNA, these effects were abrogated, highlighting the need for GHRS-1a signaling in these anti-inflammatory and anti-apoptotic responses (116). In a CLP-induced Sprague Dawley rat sepsis model, ghrelin treatment improved survival and reduced peritoneal bacterial load and pulmonary TNF-α and IL-6. Signaling *via* the GSHR-1a receptor was responsible for improving mortality. Additionally, the CLP-treated group without ghrelin administration had significantly lower ghrelin content in the lung (117). A more recent study also noted that in the lung with elastase-induced emphysema, ghrelin polarized alveolar macrophages to the M2 phenotype, decreased keratinocyte-derived chemokine (a mouse IL-8 analogue), TGF-β, and TNF-α, increased IL-10 levels, and promoted lung tissue recovery in C57BL/6 mice (118). Exogenous administration of ghrelin reduced pulmonary hypertension in a monocrotaline-induced mouse model (119). Ghrelin may play a key role in altering the immune response, possibly linking gastro-intestinal tract function and metabolism to immunity in the lung. Further, studying the effects of ghrelin receptor agonists on the lung may provide an important avenue for host-directed therapy during lung infection and inflammatory conditions.

## Pancreatic Hormones

### C-Peptide

C-peptide is a 31 amino acid peptide that links the insulin A and B subunits and is cleaved from proinsulin to form insulin. Despite evidence of C-peptide–induced intracellular signaling activity in KATOII cells (a human gastric tumor cell line), a specific receptor for C-peptide has been elusive and a proposed receptor, GPR146, remains contentious (120, 121). However, its chemotactic properties were noted when *in vitro* experiments showed that C-peptide increases human monocyte chemotaxis in a concentration dependent manner (122). Several studies identified beneficial effects of C-peptide in the lungs. C-peptide treatment ameliorates the inflammatory response and lung inflammation resulting from hemorrhagic shock in male Wistar rats, reducing plasma IL-1, IL-6, MIP-1α, and CXCL1 (123). Jeon et al. (2019) found that C-peptide prevents vascular leakage by inhibiting VEGF-induced transglutaminase 2 activation in the lungs of streptozotocin-treated mice and human pulmonary microvascular endothelial cells (124). These findings were also corroborated in C57BL/6 mice treated with C-peptide after hemorrhagic shock and resuscitation, showing a reduction in inflammatory markers and pulmonary protein leakage (125). Furthermore, Vish et al. (2007) used a Swiss albino mouse model with LPS-induced endotoxic shock to investigate the effects of C-peptide treatment. LPS treatment in the lungs of mice reduced PPARγ gene expression, stimulated ERK1/2 activation, and induced lung injury. These effects were reversed by C-peptide treatment (126). While PPARγ has been shown to have anti-inflammatory effects, ERK1/2 activation is involved in developing a pro-inflammatory response. These findings indicate an anti-inflammatory nature of C-peptide signaling in the lung, though further studies in this field are required to determine insights in its pulmonary effects and influence during infection. The identification and characterization of the signaling mechanisms of C-peptide are key to understanding its effects and potential applications.

### Insulin

The insulin receptor (IR) is expressed in varying degrees across diverse tissues, including the lungs. In the lungs, IR gene expression predominates in endothelial cells, club cells and ciliated cells, and alveolar type 1 and type 2 cells, with lower expression in lung immune cells (84). The two IR isoforms, IR-A and IR-B, and IGF-1R bind their ligands, insulin, proinsulin, IGF-1, and IGF-2, with differing potencies (EC_50_). IR-A displays the lowest EC_50_ values for insulin, proinsulin, and IGF-2 (127). IR-B predominates in the liver and adipose tissue, while leukocytes only express the IR-A isoform (128). Different pathways are activated by the two IR isoforms (129) indicating that an altered ratio of the isoforms can alter the outcomes of insulin signaling.

GC treatment increases IR expression, while hypothyroidism induction reduces the IR expression in fetal rabbit lung, indicating the influence of other hormones on the lung’s insulin response (130). During allergic lung inflammation in rats, insulin secretion and IR expression is increased on infiltrating inflammatory cells, predominantly monocytes and macrophages (131). In an elastase-induced emphysema Wistar rat model with alloxan-induced diabetes, diabetic rats had significantly lower leukocyte infiltration into the lungs, which was significantly increased upon subcutaneous treatment with insulin (132). While suggesting that insulin treatment improves the deficient immune cell migration in diabetes, it also may indicate pro-inflammatory effects due to leukocyte recruitment, although this study did not use a vehicle treatment control to compare with the insulin treated group.

Martins et al. (2008) isolated alveolar macrophages by BAL from male Wistar rats and stimulated them with LPS to assess the inflammatory response. In the insulin treated cells after LPS-stimulation, there was a significant reduction in the LPS-induced p38 MAPK, PKC, and Akt activation, and TNF-α secretion (133). A similar study in specific-pathogen-free C57BL/6 mice with alloxan induced diabetes found that insulin treatment of alveolar macrophages decreased LPS-induced TNF-α and IL-6 production *ex vivo* (134). Thus, the response of the lung tissue to insulin is still unclear, although some studies indicate anti-inflammatory changes in gene and cytokine expression in alveolar macrophages treated with insulin, others suggest that insulin treatment promotes leukocyte migration to the lung. There is however limited information available regarding the effects of insulin resistance and hyperinsulinemia on immune function in the lung, despite numerous studies on respiratory function in these cases. Understanding these lung responses to insulin is crucial in the application of insulin treatments, including inhalants and injectables, and understanding the pathogenesis of infection in the lungs of patients with diabetes mellitus.

### Glucagon

Reports of glucagon receptor (GCGR) expression and function in the lung are scarce. While two studies suggest little to no expression of GCGR in the lungs of rats, another study suggests its predominant expression in mouse lungs, liver, kidney, adrenal glands, and stomach (135–137). Glucagon receptors have been identified in lymphocytic cells from mice and rats, and in lymphoid cell lines (138).

Nebulized glucagon delivery to the lung improves the forced expiratory volume by 22% in asthmatic patients with methacholine-induced bronchospasm (139). This indicates responsiveness of the lung smooth muscle to glucagon. It was later shown that glucagon induces smooth muscle relaxation in the lungs of male A/J mice by inducing cAMP response element binding protein (CREB), eNOS, and cyclooxygenase 1 (COX-1) activity, and the subsequent release of second messengers nitric oxide and prostaglandin E_2_ (140). In this study, glucagon treatment one hour before LPS administration limited LPS-induced lung inflammation and airway hyperreactivity, preventing increases in TNF-α levels. Unlike neutrophils, LPS-induced monocyte infiltration was not reduced by glucagon pretreatment, indicating that glucagon may not influence monocyte migration and chemotaxis. This group further assessed the anti-inflammatory properties of glucagon in ovalbumin-induced lung inflammation and airway hyperreactivity (141). Glucagon treatment prevented airway hyperreactivity and eosinophilia, and reduced IL-4, IL-5, IL-13, TNF-α, CCL11, and CCL24 levels in lung tissue.

## Adipose Hormones

### Adiponectin

As summarized in Ye et al. (2013), adiponectin affects metabolic functions, such as glucose metabolism, insulin sensitivity, oxidative stress, and inflammation, in numerous systems, including adipose tissue, B cells, and macrophages (142). The mRNA expression of adiponectin receptors AdipoR1 and AdipoR2 have been demonstrated by Northern blot in mouse and human lung tissues, although a considerably lower expression was observed in human lung tissue compared to other tissues (143). Constant adiponectin infusion (using osmotic pumps) in BALB/cJ mice reduces allergic airway inflammation and hyperresponsiveness induced by ovalbumin challenge, while the lack of adiponectin in knockout mice promotes greater allergic airway inflammation (144). Additionally, adiponectin knockout mice have a higher frequency of TNF-α producing alveolar macrophages, which is reduced upon adiponectin supplementation, and increased transcription of IL-1α, IL-6, IL-12β, IL-17, and TNF-α in their lungs during aspergillosis, than wild type mice (145).

In humans, several studies report an inverse association of serum adiponectin and asthma prevalence and severity (146). Adiponectin promotes an anti-inflammatory M2 macrophage phenotype, while inhibiting an M1 phenotype. This was observed when adiponectin deficient mice displayed a predominance of M1-polarized macrophages and fewer M2-polarized macrophages in the peritoneum and adipose tissue. Treatment with adiponectin reversed this observation, promoting an M2 phenotype in human monocyte-derived macrophages and adipose tissue cells, and reducing LPS-stimulated TNF-α, iNOS, and MCP-1 gene expression (147). Agonist therapy of the adiponectin receptor shows promise against inflammatory conditions such as systemic sclerosis, obesity-related disorders, and T2D. This was shown using the adiponectin agonist, AdipoRon, which ameliorated signs of diabetes in db/db and high fat diet fed mice, and ameliorated dermal fibrosis in mice, also promoting a Th2/Th17 immune response (148, 149). While adiponectin is an abundant adipokine and displays anti-inflammatory functions, more information is needed on whether its levels are altered to compensate for inflammation. Additionally, the applications of adiponectin agonist therapy against lung inflammatory responses require further investigation.

### Leptin

Leptin regulates innate and adaptive functions of immune cells. Leptin upregulates phagocytosis and pro-inflammatory cytokine production *via* phospholipase activation, and stimulates lineage marker expression, including HLA-DR, CD11b, and CD11c in monocytes (150). Thus, leptin is an important pro-inflammatory mediator predominantly produced in adipose tissue. Leptin receptor mRNA, including OB-Ra, -Rb, and -Re subtypes, are expressed in the mouse lung (151, 152).

Leptin receptor deficient db/db mice infected with *M. tuberculosis* have a higher lung bacterial load, disorganized granulomas, and abnormalities in lung cytokine production, with delayed IFN-γ production as compared to wild-type mice (153). Similarly, leptin deficient ob/ob mice with pulmonary infection by *Klebsiella pneumoniae*, *S. pneumoniae*, or *M. abscessus* have reduced survival (154). Suzukawa et al. (2015) used bronchial cells in *ex vivo* cultures to assess the effects of leptin stimulation on lung cells, notably these specimens were obtained from lung with localized tumors. Leptin treatment increased CCL11, G-CSF, VEGF, and IL-6 production and upregulated ICAM-1 expression, a change that coincided with activation of NF-κB, highlighting the pro-inflammatory potential of leptin and its ability to induce migration and chemotaxis of immune cells (155). This has implications for increasing the inflammatory response and leukocyte infiltration into the lung in individuals with increased leptin levels, such as during obesity. Additionally, OB-R expression was upregulated by IFN-y and IL-1β, implying that during type 1 inflammatory responses, the lung epithelial cells are more sensitive to these pro-inflammatory effects of leptin. These effects were recapitulated in later work by the same group in a lung fibroblast cell line (156).

Thus, although leptin presence may be required for a functional immune response to infection, higher levels may promote excessive inflammation. These effects require further investigation given the importance of increased leptin during obesity and T2D, which display higher prevalence of comorbid lung diseases (157).

### Lipocalin-2

Lipocalin-2, or neutrophil gelatinase associated lipocalin (NGAL), is an adipokine with broad tissue expression. Zhang et al. (2012) investigated this using tissue microarray technology across humans tissues from embryos, fetuses, neonates, and adults (158). This study demonstrated expression of NGAL and its receptor (NGALR) in nervous, renal, adrenal, pituitary, spleen, lymph node, and skin tissues, with NGALR additionally expressed in lung and pancreatic tissues in adults.

NGAL influences the immune response by polarizing macrophages to an M1 phenotype (159), and altering neutrophil chemotaxis and cytokine secretion (160). Wang et al. (2019) investigated these immune modulating capabilities of NGAL, finding defective neutrophil chemotaxis, and reduced inflammatory cytokine and chemokine production in neutrophils and macrophages in NGAL deficient mice infected with *E. coli*, and increased macrophage migration and phagocytosis upon *in vitro* NGAL treatment (161). These findings were recapitulated in pulmonary *M. tuberculosis* infection in mice, whereby NGAL promoted neutrophil recruitment and alveolar macrophage G-CSF and CXCL1 production, while reducing T cell recruitment and CXCL9 production. NGAL deficiency resulted in larger granuloma size in chronic *M. tuberculosis* infection, coinciding with elevated lung T cell and CXCL9 presence (162). Furthermore, NGAL is important in the lung mucosal immunity against *K. pneumoniae* infection, playing a role in bacterial clearance (163). This study showed that NGAL is expressed on human bronchial epithelial cells in response to *in vitro* stimulation with IL-1β, IL-17A, IL-17F, or TNF-α, and *in vivo* upon *K. pneumoniae* infection in the lungs of C57BL/6 mice in a TLR4-dependent manner *via* the MyD88 signaling pathway. TLR4 knockout and NGAL knockout mice had significantly higher lung bacterial loads at 12 hours post infection, which was reduced significantly by exogenous NGAL administration 4 hours prior to sacrifice. Expression of NGAL is critical to the immune response in the lung and demonstrates essential functions during infection.

### Resistin

Resistin is an adipokine that binds to TLR4 and channel-activating protease 1 (CAP1), a serine protease. Two studies demonstrated resistin binding to TLR4, with resistin activating TLR4 in porcine alveolar macrophages and competing with LPS for TLR4 in human monocytes (164, 165). TLR4 and CAP1 are expressed on human alveolar macrophages and type 2 alveolar cells, indicating that resistin could play a role in the lung immune response during infection (77, 166). Resistin binds to decorin and ROR1 in mice with a lower affinity, though these are rarely expressed on human monocytes and macrophages (167), ROR1 is expressed on human alveolar type 1 cells and decorin is expressed on human lung fibroblasts and endothelial cells.

Using a humanized mouse model, Jiang et al. (2014) demonstrated that resistin expression exacerbated LPS-induced lung injury *via* neutrophil recruitment to the lung during LPS treatment and promoted their activation in the lung, with increased TNF-α and MIP-2 release, and increased neutrophil extracellular trap formation (168). Additionally, resistin-like molecules (RELMs) are homologues of resistin and have immunomodulatory effects, demonstrated in lung tissue, with pro-inflammatory and tissue remodeling capabilities (169, 170). Inflammation-mediated lung injury can occur *via* activation of TLR4/NF-κB pathways (171, 172). However, further studies are necessary to assess the effects of resistin signaling on lung immune function during infection.

## Type 2 Diabetes Impacts Lung Immune-Endocrine Interactions: Implications for TB

Endocrine alterations play a crucial role in the development and progression of T2D. Alterations in hormone levels have been identified in the plasma of T2D patients, implicating endocrine signaling in the dysregulation of inflammatory responses in T2D patients (173). Hormones with suggested pro-inflammatory properties in the lung are increased in the blood of T2D patients, such as TSH, leptin, resistin, and insulin (**Table 1**). Conversely, many hormones with potential anti-inflammatory effects are reduced during T2D, such as CRH, DHEA, T4, testosterone, adiponectin, NGAL, GIP, GLP-1, and ghrelin, while ACTH, cortisol, glucagon, and C-peptide also have anti-inflammatory potential but are raised in T2D patient blood plasma. This shift toward pro-inflammatory hormone signaling in T2D may be a mechanism for the low-grade chronic inflammation in T2D patients and provides an impetus for investigating immune-endocrine alterations in the lungs of these patients. It also indicates that a complex interaction between these hormones and the immune system is at play, requiring deconvolution before useful and exploitable insights can be gained.

Under the influence of diet and GIT function, gastric hormones contribute to the control of systemic inflammatory responses. Although GIP may play a role in compensating for insulin resistance, lower GLP-1 and ghrelin may indicate a predominance of inflammatory molecules and downregulation of anti-inflammatory mechanisms. Additionally, a role for the gut microbiome in influencing the immune response *via* gastric hormones lays an intriguing path for further research into T2D immune responses.

Foamy macrophage formation, which provides a niche for mycobacterial survival and supply crucial lipid nutrients to the bacilli, is suppressed by GIP and GLP-1 (174). During T2D, GLP-1 and GIP are reduced and dyslipidemia typically presents with higher levels of triglycerides (175), which accumulate in foamy macrophages (176).

TB is a leading cause of death worldwide, with an incidence of 10 million and a mortality between 1.1 and 1.3 million in 2019 (177). T2D is a highly prevalent risk factor for increased disease severity, poor treatment outcomes, relapse, and death in TB patients, and is projected to rise from an estimated 9.3% in 2019 to 10.9% by 2045 (178). Pleural fluid of TB patients contains elevated levels of GH and cortisol compared to healthy controls (18, 179), while DHEA concentration is reportedly lower in the plasma of TB patients compared to that of healthy controls (3, 18).

Hormone levels change differently over the course of TB treatment between treatment failure and cured patients. These include cortisol, T4, and total amylin levels, which are higher in treatment failure compared to cured patients at month 6 of TB treatment, and decrease over the course of treatment in the cured patients only (1). These hormones may promote immunosuppression during treatment, reducing the function of immune cells to work synergistically with antibiotics to clear mycobacteria from the lung.

Santucci et al. (2011) and Fernandez et al. (2020) found lower leptin levels and higher cortisol levels in TB patients compared to controls, coinciding with higher inflammatory biomarkers, such as CRP, IL-6, and IL-1β (16, 31). Furthermore, Santucci et al. (2011) found leptin and cortisol changes to be more extreme in severe TB disease than in mild disease. OB gene knockout experiments in mice show that leptin is necessary for mycobacterial control and IFN-y response (180). Characterising the effects of hormones on lung homeostasis and immune responses to infection is paramount. Experiments including overexpression and knockout of hormones and their receptor may highlight such effects.

**Figure 2:**
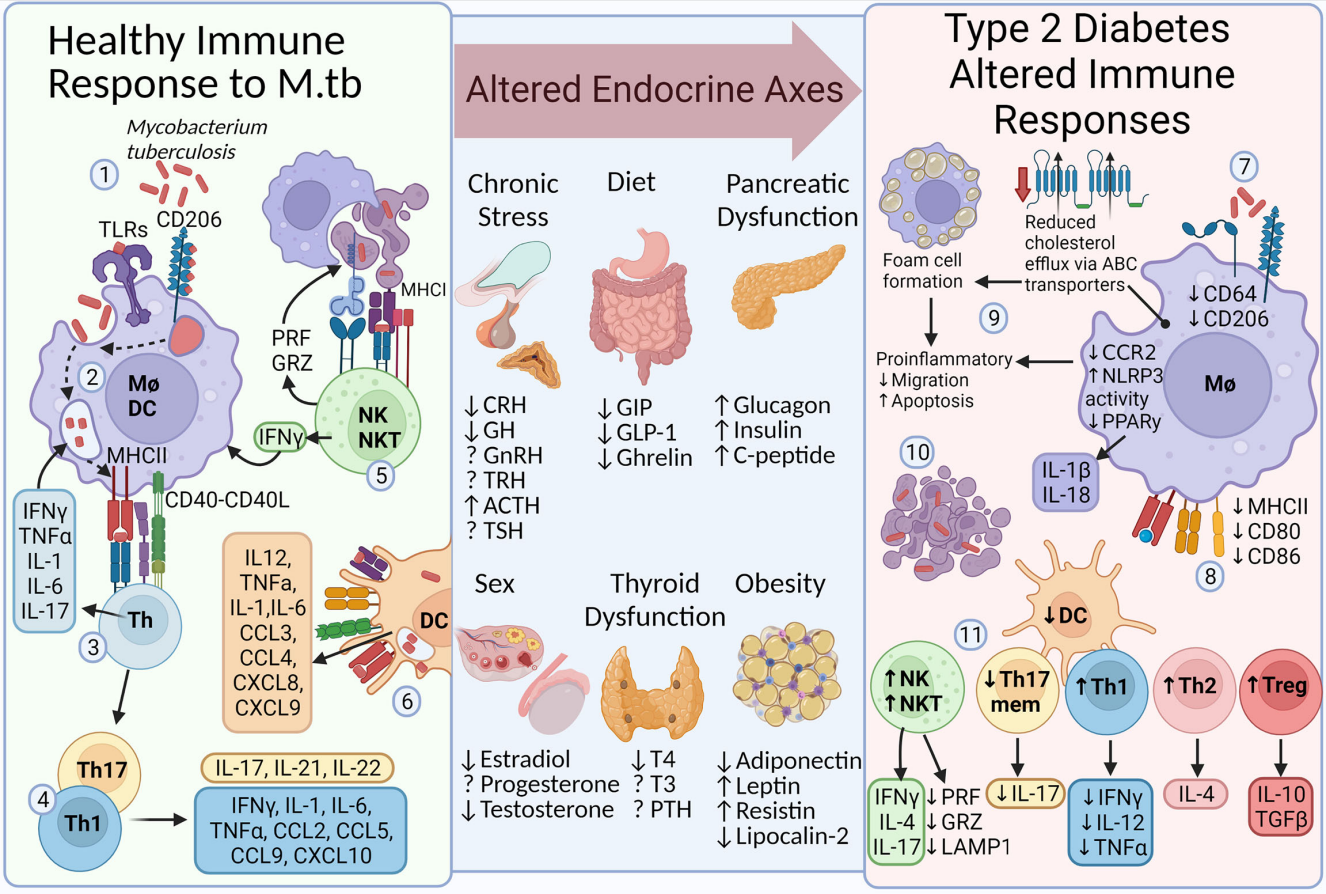
Altered endocrine signaling affects the appropriate immune response to infection. In an otherwise healthy immune system, the response to *Mycobacterium tuberculosis* includes several essential functions. **1**, PAMP-PRR interaction (e.g. TLR4 and CD206) and phagocytosis. **2**, Phagosome-lysosome fusion and presentation (MHCII). **3**, Activation of adaptive immune responses (CD40-CD40L) enhances the innate killing response *via* cytokines (e.g. IFNγ). **4**, Th1 and Th17 predominate, stimulating mycobacterial control and immune cell influx *via* cytokine and chemokine release. **5**, NK cells induce apoptosis, allowing efferocytosis by macrophages, and enhance killing by IFNγ release. **6**, DCs also promote killing, antigen presentation, and immune cell chemotaxis. The immune response is influenced by sex hormone production, diet-induced gastric hormones, stress-related responses from the brain stem, thyroid dysfunction, and the development of chronic metabolic diseases such as T2D and obesity. Thus, the immune response to *M. tuberculosis* is altered during T2D. **7**, Macrophage PRR expression (e.g. CD64 and CD206), phagocytosis, and killing is diminished. **8**, Expression of antigen presenting proteins is reduced. **9**, Expression of cholesterol efflux transporters (ABC) is reduced, leading to cholesterol accumulation and foam cell formation. Foam cells have reduced migratory capacity and increased rate of apoptosis. **10**, Reduced efferocytosis results in a cholesterol rich environment and providing a niche for mycobacterial growth. **11**, Additionally, altered ratios of Th cell subsets and dendritic cells influence cytokine levels. PAMP, pathogen-associated molecular pattern; PRR, pattern recognition receptor; TLR, toll-like receptor; MHC, major histocompatibility complex; Mφ, macrophage; DC, Dendritic cell; Th, CD4+ helper T cell; PRF, perforin; GRZ, granzyme; Treg, regulatory T cell; ABC, ATP-binding cassette cholesterol transporter; T2D, Type 2 Diabetes; TB, Tuberculosis; CRH, corticotropin-releasing hormone; GH, growth hormone; GnRH, gonadotropin-releasing hormone; TRH, thyrotropin -releasing hormone; ACTH, adrenocorticotropic hormone; T4, Thyroxine; T3, triiodothyronine; TSH, thyroid-stimulating hormone; PTH, parathyroid hormone; GIP, gastric insulinotropic peptide; GLP-1, glucagon-like peptide 1; TSH, thyroid-stimulating hormone; DHEA, dehydroepiandrosterone; This figure was created with BioRender.com.

The immune response to infection, such as during TB, involves recognition and phagocytosis of pathogens by macrophages and dendritic cells, killing by phagolysosome fusion, and presentation on major histocompatibility complexes allowing the activation of adaptive immune responses (181–183). However, T2D alters the immune response to *M. tuberculosis*, having reduced pathogen recognition, phagocytosis, killing, and presentation due to reduced pattern recognition receptor and major histocompatibility complex expression on macrophages. Altered macrophage lipid trafficking results in the formation of foam cells, with reduced phagocytic function and increased apoptosis, a cholesterol rich environment develops, providing nutrients to persisting mycobacteria. Lymphocytes dictate the developing immune response and altered lymphocyte levels in T2D include higher anti-inflammatory responses (Th2 and Treg) and reduced pathogen specific memory Th17 and Th1 responses, additionally natural killer cells are increased but their responses are reduced (184–186). The influence of hormones in determining these altered immune responses requires further investigation and their alteration during T2D provides a useful model for these altered immune responses (**Figure 2**).

Clinically, T2D delays TB treatment response, while also increasing the risk of poor TB treatment outcomes, relapse, and death (187–189). In addition, more severe presentations of TB are seen in T2D patients (190). Comorbid T2D alters TB and latent TB infection adipokine levels, with the pro-inflammatory adipokine leptin levels increased, while anti-inflammatory adiponectin levels decreased compared to those of TB patients without T2D (23), Kumar et al. (2016) found this change to be independent of BMI (23, 31). However, others have found opposite results, for example, Zheng et al. (2013) found higher leptin levels in TB patients and lower levels in TB-T2D patients. This result is unexpected, since wasting during TB disease is usually accompanied by decreased adiposity and leptin levels (26). This study also found lower ghrelin levels in TB patients with and without T2D. These disparities may be due to different genetic and socio-economic backgrounds within the study populations. Clarity regarding these hormones may require larger studies in participants from diverse genetic backgrounds.

## Discussion

Endocrine alterations contribute to changes in immune responses. While some hormones have been studied extensively for their immune-regulatory properties, few have been sufficiently investigated in the lung immune response to infection and compared to their effects outside the lung. Fewer still have been assessed in the lungs during T2D, a major endocrine dysregulating disease, and TB, a bacterial lung disease. While we have discussed more defined hormones in this review, others including progesterone, thyroid-stimulating hormone, parathyroid hormone, epinephrine, norepinephrine, melotonin, adipsin, require further investigation. Additionally, while some evidence suggests that receptors for hormones are not expressed in the lung tissue, such as C-peptide and ghrelin, other studies have described responses of lung tissue or immune cells to these hormones, indicating that alternative pathways of stimulation may be responsible for these effects or more sensitive methods are required for their evaluation in lung tissue. Elucidating the role of these hormones in modulating immune cell function in the lung will bridge a critical gap in our understanding of immune responses in the lungs.

Many studies thus far have investigated hormone-induced cellular responses in cell lines, peripheral blood cells, and in mouse and rat models. Cells under specific environmental conditions respond to hormonal stimulation differently. Thus, interpretation of these studies must be considered according to the cellular compartment or animal model they were performed in. While providing informative results, the relevance of these results to disease in the human lung remains to be validated with studies based on the human lung. Future studies assessing endocrine driven immune responses in the lung should incorporate human tissues or cells, like lung biopsies or resections and BALF, to build on current knowledge in this field with more relevance to human disease.

Understanding the factors contributing to the development, progression, and treatment response of lung diseases is crucial for improving the avenues of treatment, such as host-directed therapies, especially since antibiotics in development are deemed a short-term solution needing supplementation by other means of treatment (191). Hormone receptors may provide improved adjunctive therapeutic targets, given an improvement in our understanding of their functions in the lung under disease.

## Author Contributions

TW and LK conceived the idea for the review. TW, LK, and KR reviewed the current literature. TW and LK wrote the manuscript. TW, LK, MCS, and KR critically reviewed the manuscript.

## Funding

This study was supported by the National Institutes of Health (NIH), National Institute of Allergy and Infectious Diseases (NIAID) and the South African Medical Research Council under the US-South African Program for Collaborative Biomedical Research (grant number R01AI116039). TW was funded by the National Research Foundation of South Africa. The content is solely the responsibility of the authors and does not necessarily represent the official views of any of the funding agencies.

## Conflict of Interest

The authors declare that the research was conducted in the absence of any commercial or financial relationships that could be construed as a potential conflict of interest.

## Publisher’s Note

All claims expressed in this article are solely those of the authors and do not necessarily represent those of their affiliated organizations, or those of the publisher, the editors and the reviewers. Any product that may be evaluated in this article, or claim that may be made by its manufacturer, is not guaranteed or endorsed by the publisher.
